# Multidrug-resistant organisms may be associated with bed allocation and utilization efficiency in healthcare institutions, based on national monitoring data from China (2014–2020)

**DOI:** 10.1038/s41598-023-49548-6

**Published:** 2023-12-12

**Authors:** Xing-Tian Wang, Hua Meng, Dong-Feng Pan, Xiao-Yu Zheng, Wen-Wen Lu, Chen Chen, Ming Su, Xin-Ya Su, Zhuo Liu, Xiao-Juan Ma, Pei-Feng Liang

**Affiliations:** 1 Department of Medicine Statistics, People’s Hospital of Ningxia Hui Autonomous Region, Yinchuan, 750002 Ningxia Hui Autonomous Region China; 2 School of Public Health and Management, Ningxia Medical University, Yinchuan, 750004 Ningxia Hui Autonomous Region China; 3 Department of Emergency Medicine, People’s Hospital of Ningxia Hui Autonomous Region, Yinchuan, 750002 Ningxia Hui Autonomous Region China; 4 Ningxia Chinese Medicine Research Center, Yinchuan, 750021 Ningxia Hui Autonomous Region China; 5 Yinchuan Stomatology Hospital, Yinchuan, 750002 Ningxia Hui Autonomous Region China

**Keywords:** Risk factors, Health services

## Abstract

Analyzing the influence of the bed allocation and utilization efficiency in healthcare institutions on the isolation proportion of Multidrug-resistant organisms (MDROs) to provide data to support prevention and control of MDROs. In this study, the provincial panel data from 2014 to 2020 in China on health resource indicators, including the number of beds per 1,000 population, hospital bed utilization rate, and average hospital stay from 2014 to 2020 in China were used to analyze the relationship between bed allocation or utilization efficiency and MDROs by the panel data quantile regression model. It was shown that the number of beds per 1,000 population had a negative effect on the isolation proportion of methicillin-resistant *Staphylococcus aureus*, vancomycin-resistant *Enterococcus faecalis*, vancomycin-resistant *Enterococcus faecium*, penicillin-resistant *Streptococcus pneumoniae*, methicillin-resistant coagulase-negative *Staphylococcus*, and cefotaxime or ceftriaxone resistant *Escherichia coli* (regression coefficient < 0, *P* < 0.05). The utilization rate of hospital bed had a positive effect on the isolation proportion of methicillin-resistant *Staphylococcus aureus*, methicillin-resistant coagulase-negative *Staphylococcus*, vancomycin-resistant *Enterococcus faecium*, penicillin-resistant *Streptococcus pneumoniae*, cefotaxime or ceftriaxone resistant *Escherichia coli*, carbapenem-resistant *Escherichia coli*, cefotaxime or ceftriaxone resistant *Klebsiella pneumoniae*, carbapenem-resistant *Klebsiella pneumoniae*, carbapenem-resistant *Pseudomonas aeruginosa*, and carbapenem-resistant *Acinetobacter baumannii* (regression coefficient > 0, *P* < 0.05). The average hospital stay had a positive effect on the isolation proportion for several antibiotic-resistant organisms, including methicillin-resistant *Staphylococcus aureus*, methicillin-resistant coagulase-negative *Staphylococcus*, vancomycin-resistant *Enterococcus faecalis*, vancomycin-resistant *Enterococcus faecium*, penicillin-resistant *Streptococcus pneumoniae*, cefotaxime or ceftriaxone resistant *Escherichia coli*, carbapenem-resistant *Escherichia coli*, quinolone-resistant *Escherichia coli*, cefotaxime or ceftriaxone resistant *Klebsiella pneumoniae*, carbapenem-resistant *Pseudomonas aeruginosa*, and carbapenem-resistant *Acinetobacter baumannii* (regression coefficient > 0, *P* < 0.05). Bed allocation and utilization efficiency in healthcare institutions may affect the isolation proportion of MDROs in varying degrees.

## Introduction

The widespread use of antibiotics has inevitably led to the emergence of drug resistance. Multidrug-resistant organisms (MDROs)^1^, which are defined as organisms that have acquired non-susceptibility to at least one agent in three or more antimicrobial categories, present a significant challenge in terms of drug selection, infection prevention, and control. It has become one of the most paramount health perils in the twenty-first century^2^. Although some data suggest partial success in the prevention and control of hospital-associated MDROs infections, it is impossible to ignore^3^, the relatively high risk of death in patients with MDRO infections, which is 64% higher than in patients with non-drug-resistant infections. This highlights the importance of preventing and controlling the transmission of MDROs^4^.

Hand hygiene, screening, and isolation measures are standardized to interrupt the transmission of MDROs and prevent nosocomial infections in healthcare institutions^5–9^. Recent studies have found that overcrowding in hospitals and a lack of sufficient medical staff increase the spread of MDROs^10–16^. This is likely due to decreased compliance with hand hygiene, increased movement of patients and staff between hospital wards, and the overwhelming demand on screening and isolation facilities^17^. High bed occupancy and high patient-to-nurse ratios can increase the occurrence of adverse patient safety events. In urban teaching hospitals with high occupancy rates in the United States, there was a 15% increase in adverse event rates for every 10% increase in bed occupancy rate, and a 28% increase for every 0.1% increase in the patient-to-nurse ratio^18^.

Bed allocation and utilization efficiency refers to the number of beds owned by healthcare institutions and the proportion of beds that are actually being used. Some observational studies have analyzed the relationship between hospital bed utilization and MDROs. These studies indicate that overcrowding and high bed occupancy may be relevant factors in the spread of MDROs within hospitals^19–26^. In this study, we utilized provincial panel data from China to extract three specific indicators: Number of beds per 1,000 population, Hospital bed utilization rate, and the Average hospital stay. Our objective is to examine the correlation between bed allocation and utilization efficiency and MDROs, with the intention of offering empirical evidence to aid in the prevention and control of MDROs.

## Results

### Percentage of targeted species

After eliminating duplicate isolates on the principle of retaining the first isolate of the same bacteria from the same patient, 20,262,294 bacterial isolates were included for analysis from 2014 to 2020. The eight targeted bacterial species in our study accounted for 68.9% of all isolates included in CARSS surveillance. *Escherichia coli* was the most common targeted species identified (20.7% of isolates), followed by *Klebsiella pneumoniae* (14.3%), *Staphylococcus aureus* (9.4%), *Pseudomonas aeruginosa* (8.8%), and *Acinetobacter baumannii* (7.2%), *Enterococcus faecium* (2.8%), *Enterococcus faecalis* (2.8%), and *Streptococcus pneumoniae* (2.8%). There were no changes in the proportions of the bacterial species during the study period (Table 1).

**Table 1:** Constituent of targeted species, CARSS, 2014–2020.

Species	Total		2014		2015		2016		2017		2018		2019		**2020**	
	(n = 20 262 294)		(n = 2 227 420)		(n = 2 400 786)		(n = 2 727 605)		(n = 2 894 517)		(n = 3 234 372)		(n = 3 528 471)		(n = 3 249 123)	
	n	%	n	%	n	%	n	%	n	%	n	%	n	%	n	%
Gram-positive bacteria	5,917,700	29.2	634,414	28.5	695,066	28.9	794,073	29.1	859,388	29.7	952,023	29.4	1,043,535	29.6	939,201	28.9
*Staphylococcus aureus*	1,901,629	9.4	194,749	8.7	223,674	9.3	256,716	9.4	273,872	9.5	309,801	9.6	337,039	9.6	305,778	9.4
*Enterococcus faecium*	569,071	2.8	55,769	2.5	61,920	2.6	73,469	2.7	78,444	2.7	91,788	2.8	105,437	3	102,244	3.1
*Enterococcus faecalis*	575,526	2.8	63,566	2.9	67,398	2.8	76,664	2.8	81,403	2.8	90,196	2.8	98,418	2.8	97,881	3
*Streptococcus pneumoniae*	574,317	2.8	61,770	2.8	64,798	2.7	72,293	2.7	84,374	2.9	101,534	3.1	113,136	3.2	76,412	2.4
Gram-negative bacteria	14,344,594	70.8	1,593,006	71.5	1,705,720	71.1	1,933,532	70.9	2,035,129	70.3	2,282,349	70.6	2,484,936	70.4	2,309,922	71.1
*Acinetobacter baumannii*	1,457,423	7.2	171,662	3.2	183,124	7.6	208,689	7.7	207,046	7.2	227,091	7.1	239,890	6.8	219,921	6.8
*Escherichia coli*	4,202,679	20.7	465,136	20.9	509,862	21.2	575,494	21.1	597,909	20.7	660,261	20.4	707,968	20.1	686,049	21.1
*Klebsiella pneumoniae*	2,889,256	14.3	308,951	13.9	336,738	14	381,198	14	411,487	14.2	465,322	14.4	503,230	14.3	482,330	14.8
*Pseudomonas aeruginosa*	1,785,500	8.8	202,817	9.1	219,558	9.1	246,242	9	253,083	8.7	283,222	8.8	299,318	8.5	281,260	8.7

### Distribution of MDROs in different years and provinces

As shown in Table 2, among gram-positive bacteria, the isolation proportion of MRSA exhibited a gradual decline, decreasing from 36% in 2014 to 29.4% in 2020. The isolation proportion of MRCNS also gradually decreased, from 79.8% in 2014 to 74.7% in 2020. The isolation proportion of VREA decreased from 0.8% in 2014 to 0.2% in 2020. Similarly, the isolation proportion of VREM decreased from 2.9% in 2014 to 1% in 2020. The isolation proportion of PRSP decreased from 4.3% in 2014 to 0.9% in 2020. In contrast, the isolation proportion of ERSP showed an increasing trend, rising from 94% in 2014 to 96% in 2020.

**Table 2:** Isolation rate of multidrug-resistant organisms in China, 2014–2020 (%).

	2014	2015	2016	2017	2018	2019	2020	Mann–Kendall test		
								Trend	Z value	*P* value
*MRSA*	36	35.8	34.4	32.2	30.9	30.2	29.4	Down	− 3.0038	0.002667*
*MRCNS*	79.8	79.4	77.5	76	75.7	75.4	74.7	Down	− 3.0038	0.002667*
*VREA*	0.8	0.8	0.6	0.4	0.3	0.2	0.2	Down	− 2.7665	0.005666*
*VREM*	2.9	2.9	2	1.4	1.4	1.1	1	Down	− 2.7665	0.005666*
*PRSP*	4.3	4.2	3.9	2.7	1.8	1.6	0.9	Down	− 3.0038	0.002667*
*ERSP*	94	91.5	94.4	95	95.4	95.6	96	Up	2.7034	0.006864*
*CTX/CRO-R ECO*	59.7	59	56.6	54.2	53	51.9	51.6	Down	− 3.0038	0.002667*
*CR-ECO*	1.9	1.9	1.5	1.5	1.5	1.7	1.6	Down	− 0.63511	0.5254
*QNR-ECO*	54.3	53.5	52.9	51	50.8	50.6	50.7	Down	− 2.7034	0.006864*
*CTX/CRO-R KPN*	36.9	36.5	34.5	33	32.4	31.9	31.1	Down	− 3.0038	0.002667*
*CR-KPN*	6.4	7.6	8.7	9	10.1	10.9	10.9	Up	2.8863	0.003898*
*CR-PAE*	25.6	22.4	22.3	20.7	19.3	19.1	18.3	Down	− 3.0038	0.002667*
*CR-ABA*	57	59	60	56.1	56.1	56	53.7	Down	− 1.9748	0.04829*

*: *P* < 0.05. MRSA: methicillin-resistant *Staphylococcus aureus*, MRCNS: Methicillin-resistant coagulase-negative *Staphylococcus*, VREA: vancomycin-resistant *Enterococcus faecalis*, VREM: vancomycin-resistant *Enterococcus faecium*, PRSP: penicillin-resistant *Streptococcus pneumoniae*, ERSP: erythromycin-resistant *Streptococcus pneumoniae*, CTX/CRO-R ECO: cefotaxime or ceftriaxone resistant *Escherichia coli*, CR-ECO: carbapenem-resistant *E. coli*, QNR-ECO: quinolone-resistant *Escherichia coli*, CTX/CRO-R KPN: cefotaxime or ceftriaxone resistant *Klebsiella pneumoniae* , CR-KPN: carbapenem-resistant *Klebsiella pneumoniae*, CR-PAE: carbapenem-resistant *Pseudomonas aeruginosa*, CR-ABA: carbapenem-resistant *Acinetobacter baumannii.*

Among the gram-negative bacteria, the isolation proportion of CTX/CR-R ECO exhibited a gradual decline, decreasing from 59.7% in 2014 to 51.6% in 2020. CR-ECO exhibited a consistently low level of isolation with an isolation rate ranging from 1.9 to 1.6%. Additionally, the isolation proportion of QNR-ECO showed a gradual decline over time, decreasing from 54.3% in 2014 to 50.7% in 2020. The isolation proportion of CTX/CR-R KPN exhibited a gradual decline from 36.9% in 2014 to 31.1% in 2020. The isolation proportion of CR-KPN has shown a consistent increase, rising from 6.4% in 2014 to 10.9% in 2020. The isolation proportion of CR-PAE exhibited a consistent decline, decreasing from 25.6% in 2014 to 18.3% in 2020. The isolation proportion of CR-ABA exhibited fluctuations ranging from 53.7 to 60%, consistently maintaining a high level. An analysis description of the isolation proportions of different MDROs is presented in Supplementary Table 1. The detection proportion of certain isolates was found to deviate across different years.

**Figure 1:**
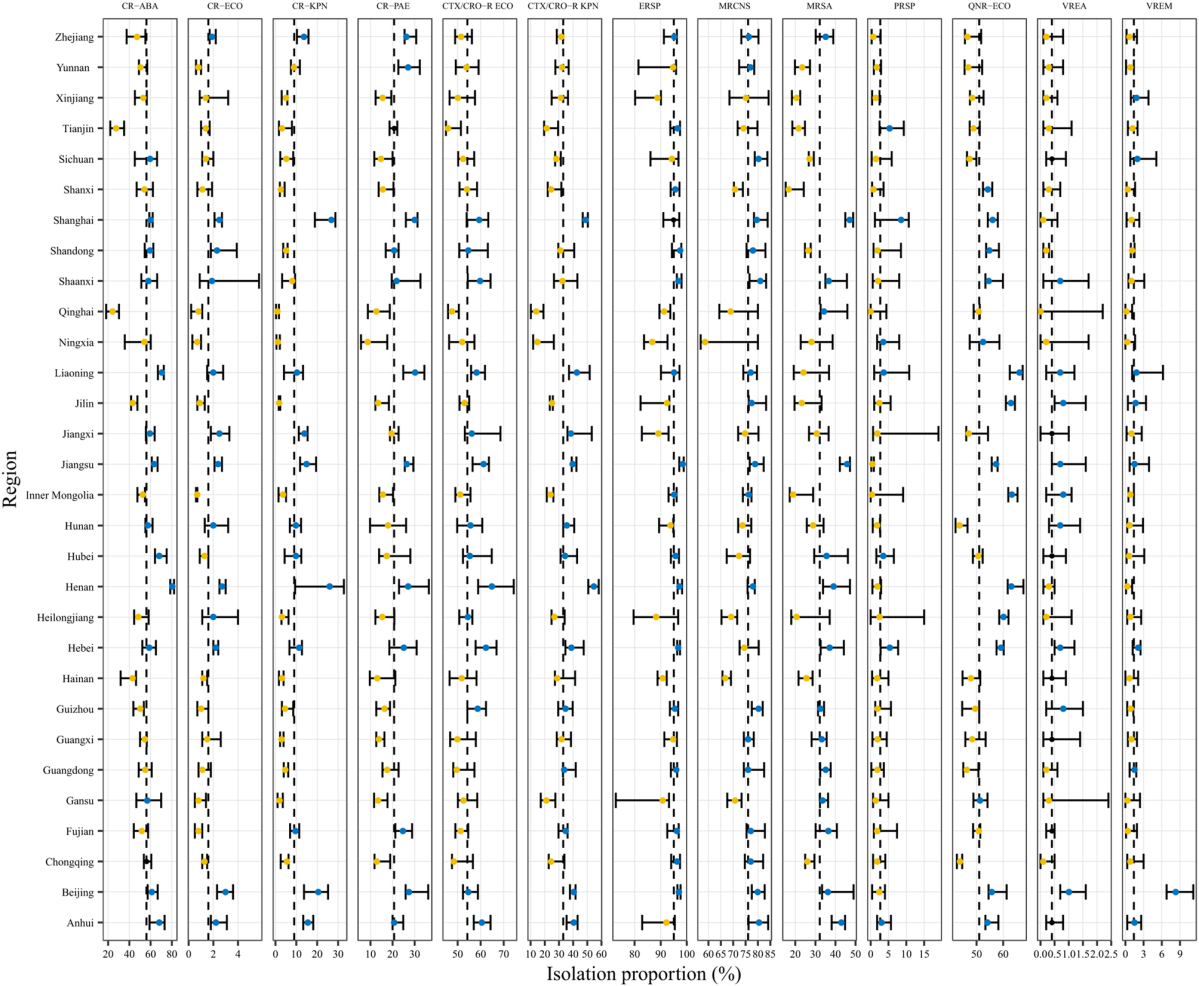
The isolation proportion of an independent Multidrug-resistant organism isolate in various provinces. Each box represents the isolation rate of an independent Multidrug-resistant organism isolates. The horizontal coordinate is isolation proportion and the vertical coordinate is different provinces. The vertical dotted line is the national median of the isolation proportion of a certain type of isolate. The dots are the median of the isolation proportion of each province from 2014 to 2020.The yellow dots represent that the isolation proportion of drug resistant bacteria in a province is lower than the national median. while the blue dots represent that the isolation rate is greater than the national median. The error bars show the range of the MDRO isolation proportion. CR-ABA: carbapenem-resistant Acinetobacter baumannii, CR-ECO: carbapenem-resistant E. coli, CR-KPN: carbapenem-resistant Klebsiella pneumoniae, CR-PAE: carbapenem-resistant Pseudomonas aeruginosa, CTX/CRO-R ECO: cefotaxime or ceftriaxone resistant Escherichia coli, CTX/CRO-R KPN: cefotaxime or ceftriaxone resistant Klebsiella pneumoniae, ERSP: erythromycin-resistant Streptococcus pneumoniae, MRCNS: Methicillin-resistant coagulase-negative Staphylococcus, MRSA: methicillin-resistant Staphylococcus aureus, PRSP: penicillin-resistant Streptococcus pneumoniae, QNR-ECO: quinolone-resistant Escherichia coli, VREA: vancomycin-resistant Enterococcus faecalis, VREM: vancomycin-resistant Enterococcus faecium.

The isolation proportions of MDROs vary across different provinces. For instance, the isolation proportion of MRSA in Shanghai, Shaanxi, Jiangsu, Beijing, and Anhui between 2014 and 2020 exceeded the national average values of 32.35%. Conversely, Yunnan, Xinjiang, Tianjin, Sichuan, and Shandong had isolation proportions below the national average. Meanwhile, there was a significant variation in the isolation proportion of MRSA in Heilongjiang and Liaoning. The distribution of other MDROs identified in various regions is illustrated in Fig. 1.

### Number of beds per 1,000 population, hospital bed utilization rate and average hospital stay in healthcare institutions in various provinces

**Figure 2:**
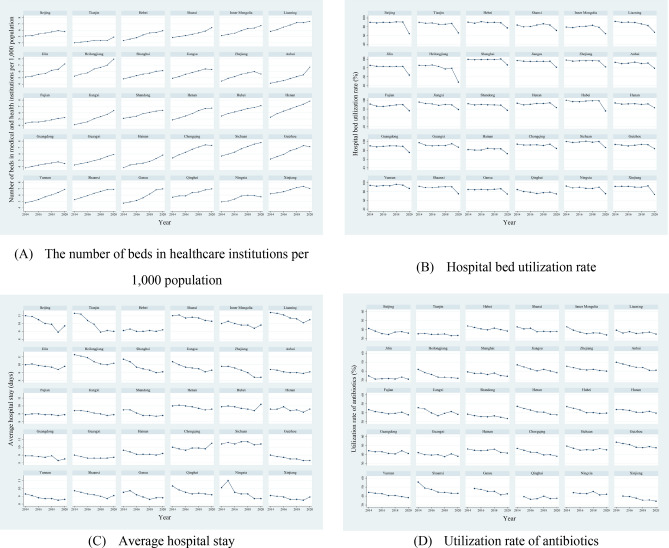
Bed allocation and utilization efficiency, utilization rate of antibiotics in healthcare institutions in various provinces. (**A**) The number of beds in healthcare institutions per 1,000 people; (**B**) Hospital bed utilization rate; (**C**) Average hospital stay; (**D**) Utilization rate of antibiotics. Note: Utilization rate of antibiotics in Gansu, Qinghai, Ningxia, and Xinjiang in 2014 were not available.

As shown in Fig. 2, the number of beds in health institutions per 1,000 population increased annually in the provinces. The hospital bed utilization rate declined more significantly in 2020 compared other years, where it remained relatively stable. The average hospital stay varied across provinces, and there was a gradual decline in the utilization rate of antibiotics. The statistical descriptions of the number of beds per 1,000 population, hospital bed utilization rate, and average hospital stay are shown in Supplementary Table 2.

### Panel quantile regression of MDROs isolation proportions and number of beds per 1,000 population, hospital bed utilization rate and average hospital stay

From the number of beds per 1,000 population on five quantile points shown in Table 3, significance at the 0.05 level was observed for the following MDROs: MRSA (τ = 0.1, 0.3, 0.5, 0.7, and 0.9), VREA (τ = 0.1, 0.3, 0.5, 0.7, and 0.9), VREM (τ = 0.1, 0.3, 0.5, and 0.7), PRSP (τ = 0.1, 0.3, 0.5, 0.7, and 0.9), MRCNS (τ = 0.7 and 0.9), CTX/CRO-R ECO (τ = 0.5 and 0.9), CTX/CRO-R KPN (τ = 0.3, 0.5, and 0.9), and CR-PAE (τ = 0.1, 0.3, and 0.9). The regression coefficients were all lower than 0, indicating that the isolation proportions of the eight MDROs mentioned above were negatively influenced by the number of beds available per 1,000 population.

**Table 3:** Association between the isolation rate of multidrug-resistant organisms and Number of beds per 1,000 population, Hospital bed utilization rate and Average hospital stay in healthcare institutions with the panel quantile regression model.

Variable	Quantile 0.1	Quantile 0.3	Quantile 0.5	Quantile 0.7	Quantile 0.9
MRSA					
Constant	− 0.820 (− 0.06)	13.070 (1.02)	11.370 (0.72)	− 3.957 (− 0.23)	− 7.910 (− 0.55)
Number of beds per 1,000 population	− 1.519** (− 1.98)	− 1.405** (− 1.98)	− 2.328*** (− 2.66)	− 2.619*** (− 2.75)	− 1.521* (− 1.90)
Hospital bed utilization rate	0.187** (2.02)	0.358*** (4.18)	0.311*** (2.95)	0.356*** (3.11)	0.397*** (4.11)
Average hospital stay	− 0.983 (− 1.02)	− 2.142** (− 2.40)	− 0.366 (− 0.33)	2.059* (1.72)	2.799*** (2.79)
Utilization rate of antibiotics	0.612*** (6.07)	0.267*** (2.86)	0.232** (2.02)	0.099 (0.79)	− 0.053 (− 0.50)
Pseudo R^2^	0.1673	0.1576	0.1245	0.1070	0.1737
MRCNS					
Constant	33.410* (1.65)	31.230*** (3.43)	51.440*** (6.84)	62.600*** (9.65)	64.990*** (8.86)
Number of beds per 1,000 population	− 0.096 (− 0.09)	− 0.289 (− 0.57)	− 0.683 (− 1.64)	− 0.945*** (− 2.63)	− 0.888** (− 2.18)
Hospital bed utilization rate	0.449*** (3.33)	0.419*** (6.89)	0.234*** (4.66)	0.147*** (3.40)	0.180*** (3.68)
Average hospital stay	0.260 (0.19)	1.041 (1.64)	0.933* (1.78)	0.566 (1.26)	0.470 (0.92)
Utilization rate of antibiotics	− 0.115 (− 0.78)	− 0.055 (− 0.83)	− 0.009 (− 0.16)	0.076 (1.62)	0.008 (0.16)
Pseudo R2	0.1412	0.1497	0.1279	0.1299	0.1292
VREA					
Constant	− 0.661** (− 2.13)	− 1.138*** (− 2.62)	− 1.413** (− 2.16)	− 1.031 (− 1.22)	1.454 (0.84)
Number of beds per 1,000 population	− 0.089*** (− 5.18)	− 0.069*** (− 2.85)	− 0.085** (− 2.34)	− 0.147*** (− 3.15)	− 0.333*** (− 3.47)
Hospital bed utilization rate	0.000 (0.21)	0.003 (1.21)	0.001 (0.34)	0.001 (0.26)	0.000 (0.00)
Average hospital stay	0.083*** (3.84)	0.112*** (3.72)	0.184*** (4.06)	0.254*** (4.34)	0.212* (1.76)
Utilization rate of antibiotics	0.012*** (5.21)	0.010*** (3.13)	0.011** (2.21)	− 0.001 (− 0.10)	− 0.011 (− 0.87)
Pseudo R2	0.0935	0.1056	0.0974	0.1146	0.1453
VREM					
Constant	− 1.921* (− 1.74)	− 2.749** (− 2.24)	− 3.627** (− 2.47)	− 5.736** (− 2.13)	− 16.650* (− 1.74)
Number of beds per 1,000 population	− 0.173*** (− 2.82)	− 0.288*** (− 4.23)	− 0.312*** (− 3.83)	− 0.299** (− 2.00)	− 0.119 (− 0.22)
Hospital bed utilization rate	0.014* (1.95)	0.025*** (3.07)	0.030*** (3.00)	0.035* (1.93)	0.066 (1.03)
Average hospital stay	0.222*** (2.88)	0.325*** (3.80)	0.388*** (3.80)	0.646*** (3.45)	1.656** (2.49)
Utilization rate of antibiotics	− 0.003 (− 0.38)	− 0.005 (− 0.52)	0.005 (0.47)	− 0.004 (− 0.18)	− 0.032 (− 0.46)
Pseudo R^2^	0.0622	0.1254	0.1287	0.1180	0.2037
PRSP					
Constant	− 0.349 (− 0.21)	− 0.066 (− 0.03)	− 3.603 (− 0.95)	− 5.988 (− 0.92)	− 13.220* (− 1.66)
Number of beds per 1,000 population	− 0.201** (− 2.20)	− 0.311** (− 2.40)	− 0.514** (− 2.45)	− 1.063*** (− 2.94)	− 1.503*** (− 3.41)
Hospital bed utilization rate	0.029*** (2.63)	0.030* (1.92)	0.032 (1.27)	0.054 (1.23)	0.070 (1.32)
Average hospital stay	− 0.093 (− 0.81)	− 0.033 (− 0.20)	0.593** (2.25)	1.118** (2.46)	2.250*** (4.07)
Utilization rate of antibiotics	0.012 (0.98)	0.020 (1.15)	0.010 (0.35)	0.015 (0.32)	0.016 (0.28)
Pseudo R^2^	0.0514	0.0568	0.0592	0.1177	0.2083
ERSP					
Constant	91.980*** (4.70)	79.260*** (7.59)	85.120*** (15.18)	94.010*** (26.87)	91.340*** (26.03)
Number of beds per 1,000 population	0.553 (0.51)	0.789 (1.36)	0.543* (1.75)	0.213 (1.10)	0.349* (1.79)
Hospital bed utilization rate	− 0.105 (− 0.81)	0.024 (0.34)	0.038 (1.02)	− 0.006 (− 0.26)	0.029 (1.24)
Average hospital stay	− 0.121 (− 0.09)	0.530 (0.73)	0.031 (0.08)	0.149 (0.61)	0.083 (0.34)
Utilization rate of antibiotics	0.089 (0.63)	0.053 (0.70)	0.074* (1.82)	0.005 (0.21)	0.013 (0.50)
Pseudo R^2^	0.0256	0.0293	0.0240	0.0076	0.0076
CTX/CRO-R ECO					
Constant	22.600** (2.22)	17.340** (2.04)	12.900 (1.59)	7.972 (0.71)	43.780*** (3.73)
Number of beds per 1,000 population	− 0.444 (− 0.79)	− 0.755 (− 1.60)	− 1.154** (− 2.57)	− 0.754 (− 1.22)	− 2.526*** (− 3.88)
Hospital bed utilization rate	0.087 (1.28)	0.149*** (2.62)	0.234*** (4.31)	0.264*** (3.53)	0.073 (0.93)
Average hospital stay	1.350* (1.91)	1.958*** (3.31)	1.909*** (3.38)	2.127*** (2.74)	2.125*** (2.60)
Utilization rate of antibiotics	0.213*** (2.88)	0.191*** (3.09)	0.242*** (4.09)	0.250*** (3.07)	0.138 (1.61)
Pseudo R^2^	0.1212	0.1273	0.1509	0.1456	0.1804
CR-ECO					
Constant	− 2.096*** (− 3.49)	− 1.476* (− 1.70)	− 1.342 (− 0.83)	− 3.092 (− 1.62)	− 5.655*** (− 2.99)
Number of beds per 1,000 population	0.054 (1.61)	0.003 (0.06)	− 0.065 (− 0.72)	− 0.105 (− 0.99)	− 0.040 (− 0.38)
Hospital bed utilization rate	0.021*** (5.22)	0.017*** (2.97)	0.026** (2.40)	0.033** (2.55)	0.023* (1.80)
Average hospital stay	0.088** (2.11)	0.107* (1.76)	0.089 (0.79)	0.262* (1.97)	0.560*** (4.26)
Utilization rate of antibiotics	− 0.003 (− 0.65)	− 0.001 (− 0.08)	− 0.002 (− 0.15)	0.007 (0.53)	0.031** (2.21)
Pseudo R^2^	0.0568	0.0382	0.0456	0.0675	0.1077
QNR-ECO					
Constant	48.300*** (6.74)	41.140*** (3.88)	29.480** (2.43)	25.080** (2.07)	9.130 (0.86)
Number of beds per 1,000 population	− 1.601*** (− 4.03)	− 0.749 (− 1.27)	− 0.476 (− 0.71)	− 0.083 (− 0.12)	0.391 (0.66)
Hospital bed utilization rate	0.016 (0.33)	− 0.109 (− 1.54)	− 0.149* (− 1.85)	− 0.081 (− 1.00)	− 0.170** (− 2.39)
Average hospital stay	0.993** (1.99)	2.710*** (3.67)	4.486*** (5.32)	4.892*** (5.79)	6.672*** (9.02)
Utilization rate of antibiotics	− 0.100* (− 1.91)	− 0.090 (− 1.16)	− 0.077 (− 0.88)	− 0.190** (− 2.15)	0.010 (0.13)
Pseudo R^2^	0.0937	0.0984	0.1445	0.1881	0.2418
CTX/CRO-R KPN					
Constant	− 18.550 (− 0.92)	16.310 (1.44)	− 0.964 (− 0.06)	− 24.350* (− 1.88)	− 45.930 (− 1.41)
Number of beds per 1,000 population	− 1.187 (− 1.06)	− 2.181*** (− 3.47)	− 2.288*** (− 2.65)	− 2.120*** (− 2.95)	− 1.232 (− 0.68)
Hospital bed utilization rate	0.623*** (4.61)	0.312*** (4.11)	0.345*** (3.31)	0.412*** (4.76)	0.476** (2.19)
Average hospital stay	− 1.838 (− 1.31)	− 1.364* (− 1.73)	0.369 (0.34)	3.157*** (3.50)	5.589** (2.47)
Utilization rate of antibiotics	0.273* (1.85)	0.254*** (3.07)	0.314*** (2.77)	0.170* (1.80)	0.025 (0.11)
Pseudo R^2^	0.1607	0.1614	0.1366	0.1431	0.1398
CR-KPN					
Constant	− 9.053** (− 2.21)	− 13.410** (− 2.13)	− 19.310* (− 1.86)	− 12.540 (− 0.77)	− 23.490 (− 0.73)
Number of beds per 1,000 population	0.391* (1.72)	0.580* (1.66)	1.099* (1.91)	0.661 (0.73)	1.677 (0.94)
Hospital bed utilization rate	0.133*** (4.86)	0.163*** (3.88)	0.273*** (3.95)	0.331*** (3.05)	0.501** (2.34)
Average hospital stay	− 0.285 (− 1.00)	− 0.244 (− 0.56)	− 0.810 (− 1.12)	− 1.057 (− 0.93)	0.789 (0.35)
Utilization rate of antibiotics	0.003 (0.09)	0.048 (1.05)	0.079 (1.05)	0.000 (0.00)	− 0.508** (− 2.17)
Pseudo R^2^	0.0606	0.0741	0.0798	0.0502	0.1109
CR-PAE					
Constant	− 0.489 (− 0.07)	2.804 (0.35)	− 13.890 (− 1.05)	− 30.660** (− 2.12)	− 20.760** (− 2.29)
Number of beds per 1,000 population	− 1.426*** (− 3.42)	− 2.222*** (− 5.04)	− 0.967 (− 1.32)	− 0.768 (− 0.96)	− 1.205** (− 2.40)
Hospital bed utilization rate	0.165*** (3.29)	0.234*** (4.40)	0.334*** (3.78)	0.357*** (3.69)	0.224*** (3.70)
Average hospital stay	0.966* (1.85)	0.473 (0.86)	0.948 (1.03)	2.595** (2.58)	3.050*** (4.83)
Utilization rate of antibiotics	− 0.054 (− 0.99)	0.023 (0.40)	0.012 (0.13)	0.0612 (0.58)	0.171** (2.59)
Pseudo R^2^	0.0862	0.1080	0.1148	0.1449	0.2006
CR-ABA					
Constant	− 79.000* (− 1.96)	− 35.910* (− 1.91)	− 22.860 (− 1.52)	− 16.570 (− 1.12)	− 33.460 (− 1.08)
Number of beds per 1,000 population	2.703 (1.21)	1.536 (1.47)	0.581 (0.70)	− 0.292 (− 0.36)	− 0.098 (− 0.06)
Hospital bed utilization rate	1.048*** (3.90)	0.602*** (4.79)	0.534*** (5.32)	0.302*** (3.05)	0.252 (1.22)
Average hospital stay	− 0.076 (− 0.03)	2.409* (1.84)	2.498** (2.39)	4.818*** (4.68)	7.174*** (3.34)
Utilization rate of antibiotics	0.298 (1.02)	0.053 (0.38)	0.100 (0.92)	0.143 (1.33)	0.241 (1.07)
Pseudo R^2^	0.1849	0.1180	0.1120	0.1060	0.1460

*p < 0.05, ** *p* < 0.01, *** *p* < 0.001, The value is “Regression coefficients” in table and “*t*” in brackets. MRSA: methicillin-resistant *Staphylococcus aureus*, MRCNS: Methicillin-resistant coagulase-negative *Staphylococcus*, VREA: vancomycin-resistant *Enterococcus faecalis*, VREM: vancomycin-resistant *Enterococcus faecium*, PRSP: penicillin-resistant *Streptococcus pneumoniae*, ERSP: erythromycin-resistant *Streptococcus pneumoniae*, CTX/CRO-R ECO: cefotaxime or ceftriaxone resistant *Escherichia coli*, CR-ECO: carbapenem-resistant *E. coli*, QNR-ECO: quinolone-resistant *Escherichia coli*, CTX/CRO-R KPN: cefotaxime or ceftriaxone resistant *Klebsiella pneumoniae*, CR-KPN: carbapenem-resistant *Klebsiella pneumoniae*, CR-PAE: carbapenem-resistant *Pseudomonas aeruginosa*, CR-ABA: carbapenem-resistant *Acinetobacter baumannii*.

Regarding the hospital bed utilization rate, statistical significance was observed at the 0.05 level for various MDROs. These include MRSA (τ = 0.1, 0.3, 0.5, 0.7, and 0.9), MRCNS (τ = 0.1, 0.3, 0.5, 0.7, and 0.9), VREM (τ = 0.1, 0.3, 0.5, and 0.7), PRSP (τ = 0.1 and 0.3), CTX/CRO-R ECO (τ = 0.3, 0.5, and 0.7), CR-ECO (τ = 0.1, 0.3, 0.5, 0.7, and 0.9), CTX/CRO-R KPN (τ = 0.1, 0.3, 0.5, 0.7, and 0.9), CR-KPN (τ = 0.1, 0.3, and 0.5), CR-PAE (τ = 0.1, 0.3, 0.5, 0.7, and 0.9), and CR-ABA (τ = 0.1, 0.3, 0.5, 0.7, and 0.9). The regression coefficients for MDROs mentioned above found to be greater than 0, suggesting a positive relationship between the isolation proportion of these MDROs and by the hospital bed utilization rate.

Regarding the average hospital stay, the statistical significance was observed at the 0.05 level for various MDROs. These include MRSA (τ = 0.7 and 0.9), MRCNS (τ = 0.5), VREA (τ = 0.1, 0.3, 0.5, 0.7, and 0.9), VREM (τ = 0.1, 0.3, 0.5, 0.7, and 0.9), PRSP (τ = 0.1 and 0.3), CTX/CRO-R ECO (τ = 0.1, 0.3, 0.5, 0.7, and 0.9), CR-ECO (τ = 0.1, 0.3, 0.7, and 0.9), QNR-ECO (τ = 0.1, 0.3, 0.5, 0.7, and 0.9), CTX/CRO-R KPN (τ = 0.7 and 0.9), CR-PAE (τ = 0.1, 0.7, and 0.9), and CR-ABA (τ = 0.3, 0.5, 0.7, and 0.9). All regression coefficients exhibited values greater than 0, suggesting a positive impact of the average hospital stay on the detection proportions of the 11 MDROs mentioned above. In the supplementary analysis (Supplementary Table 3), consistent findings were obtained, suggesting the stability of the study results.

## Discussion

Factors that contribute to the occurrence and dissemination of MDROs encompass antimicrobial drug utilization, level of disinfection and isolation practices, hand hygiene, environmental cleanliness, among others. In response to these factors, China has implemented specific strategies to enhance the control of MDROs transmission within healthcare facilities since the early 2000s^27,28^. From the analysis of national surveillance data on MDROs, it is evident that the majority of MDROs, with the exception of ERSP and CR-KPN, exhibited had a declining annual trend between 2014 and 2020.

Our study revealed that there was a negative correlation between the number of beds per 1,000 population and the proportions of most MDROs. Conversely, the hospital bed utilization rate and the average hospital stay showed a positive correlation with the proportions of most MDROs in this study. This implies that there is an inverse relationship between the number of beds per 1,000 population and detection rate of MDROs. Additionally, there is a positive correlation between the hospital bed utilization rate or the average hospital stay, and detection rate of MDROs.

The hospital bed utilization rate typically surpasses 85%, resulting in potential oversights in hospital infection management due to the demanding workload of medical personnel. A notable rise in MRSA detection was observed in acute hospitals in England from 2001 to 2004, coinciding with bed occupancy rates exceeding 90%. Cunningham et al.^19^. A significant correlation was observed between bed occupancy and MRSA rates in hospitals in the United Kingdom. This finding suggests that the spread of nosocomial pathogens is more likely when occupancy exceeds 85%. The Department of Health in the Netherlands and UK has recently issued additional guidance aimed at mitigating healthcare-associated infections. This includes measures such as insuring sufficient bed availability in nursing homes, establishing hospital isolation facilities, and implementing adjustments to nurse-to-patient ratios. Health authorities in China have implemented regulations that mandate medical institutions to adhere to the approved bed capacity when admitting patients. This measure aims to prevent the transmission of infectious isolates within hospitals and to maintain a controlled hospital bed utilization rate ranging from 85 to 93%^22^.

The limited availability of beds and the consequent inability to adequately isolate patients with MDROs may serve as a significant contributing factor to the transmission of MDROs within the hospital setting^29^. According to national surveillance data, the rates of proportion for MDROs are notably high in densely populated major cities like Shanghai and Beijing. The high incidence of MDROs may be attributed to the large population, which leads to a high demand for medical services. However, the existing medical resources, including beds and medical services, are insufficient to meet the needs of many patients, exacerbating the problem of MDROs^30^.

Therefore, the allocation of beds and the efficient utilization of beds in healthcare institutions are crucial factors in ensuring effective infection prevention and control. The Department of Medical Administration should enhance the efficiency of bed utilization through the implementation of strategies such as increasing the availability of beds and allocating sufficient medical and nursing resources. Additionally efforts should be made to reduce the occurrence of MDROs among hospitalized patients and to ensure the provision of high-quality medical care. According to the department’s bed utilization index and the prevalence of nosocomial infections, patients awaiting beds will be categorized into three levels of care: light, medium, and critical. Appropriate adjustments will be implemented, such as prioritizing less severe patients for later treatment and minimizing the addition of critical patients to hospital beds whenever feasible. In order to prevent the excessive accumulation of heavy beds, it is necessary to regularly maintain available beds for light and medium-care patients, thereby minimizing the need for additional beds.

This study is subject to certain limitations. Like all ecological studies, the observed trends in our study have the potential for ecological fallacy. We may have overlooked the supplementary factors pertaining to limitations that should have been taken into account in this study, including patient demographics, hospital services, and the availability of stewardship programs and resources, among others. There exist variations in the rates of isolation of MDROs due to differences in patient demographics, variations in hospital services, and variances in the background transmission of MDROs within specific communities. Additionally, it is important to consider the potential for survey selection bias and information bias, which cannot be disregarded.

In conclusion, MDROs are associated with bed allocation and utilization efficiency in healthcare institutions. Therefore, the Department of Medical Administration should improve the efficiency of bed utilization by increasing the number of beds and allocating more medical and nursing staff, reducing the incidence of MDROs in hospitalized patients, and ensuring the overall quality of overall medical care.

## Methods

### Data sources

The isolation proportion and drug resistance of MDROs were extracted from the surveillance report on bacterial resistance provided by the China Antimicrobial Resistance Surveillance System (CARSS, http://www.carss.cn).

The establishment and organization of CARSS was initiated by the Expert Committee on Rational Use of Drugs, which was a part of the National Health Commission of the People’s Republic of China (formerly known as the Health and Family Planning Committee of China). In the year 2014, a total of 1,100 hospitals from various provinces and autonomous regions actively took part in the survey. In the year 2020, the number of hospitals increased to 1,432. Among these, approximately three-fourths (74.1–76.2%) were tertiary hospitals, while the remaining quarter (23.8–25.8%) were secondary hospitals. All monitoring possess a sufficient operational foundation for their clinical microbiology laboratories, which have obtained certification for quality control from provincial and/or national clinical testing centers, adhering to their respective internal quality control standards.

Bacterial identification and monitoring of drug resistance were carried out in accordance with the CARSS monitoring technical protocol employing standardized methods and technical training. Quality control adhered to the guidelines set forth by the Clinical and Laboratory Standards Institute (CLSI). Routine quality control procedures were conducted on a weekly basis, ensuring stability in the test conditions. In the present study, MDROs encompassed a total of 13 types of bacterial strains commonly encountered in clinical settings, including methicillin-resistant *Staphylococcus aureus* (MRSA), Methicillin-resistant coagulase-negative *Staphylococcus* (MRCNS), vancomycin-resistant *Enterococcus faecalis* (VREA), vancomycin-resistant *Enterococcus faecium* (VREM), penicillin-resistant *Streptococcus pneumoniae* (PRSP), erythromycin-resistant *Streptococcus pneumoniae* (ERSP), cefotaxime or ceftriaxone resistant *Escherichia coli* (CTX/CRO-R ECO), carbapenem-resistant *E. coli* (CR-ECO), quinolone-resistant *E. coli* (QNR-ECO), cefotaxime or ceftriaxone resistant *Klebsiella pneumoniae* (CTX/CRO-R KPN), carbapenem-resistant *Klebsiella pneumoniae* (CR-KPN), carbapenem-resistant *Pseudomonas aeruginosa* (CR-PAE), and carbapenem-resistant *Acinetobacter baumannii* (CR-ABA).

The panel data pertaining to health resource indicators were extracted from the China Health and Health Statistics Yearbook for the years from 2014 to 2020. The provided data comprises information on the number of beds in healthcare institutions per 1,000 people, the hospital bed utilization rate, and the average length of stay. The population size is the resident population, and healthcare institutions include medical institutions, including general hospitals, Chinese medicine hospitals, and specialist hospitals. The indicator known as the Number of beds per 1,000 population represents the ratio of the total number of beds available in healthcare facilities withing a specific area to 1,000 times the number of inhabitants in that area. This indicator varies across regions due to differences in economic levels, and it serves as a reflection of the allocation of beds and the accessibility of health services. The hospital bed utilization rate is a metric that measures the proportion of beds occupied in relation to the total number of beds available on a daily basis. This metric provides an evaluation of the workload experienced by hospital beds and medical personnel. The average hospital stay is a metric that presents the utilization efficiency of beds and the quality of medical treatment. It is calculated by dividing the total number of bed days occupied by discharged patients by the total number of discharges. The utilization rate of antibiotics was obtained from the Center for Antimicrobial Surveillance, an organization established in 2006 and overseen by the National Health Commission of the People’s Republic of China (formerly known as the Health and Family Planning Committee of China). In the year 2020, the center witnessed the participation of over 4,000 hospitals, with general hospitals comparing more than 60% of the total.

### Statistical analysis

In this study, the data from all provinces in China were utilized as the study sample. The time observation points spanned from 2014 to 2020, forming a balanced panel data sample. It is important to note that the Tibet Autonomous Region was excluded from the study. Isolates and percentages were utilized in the study to offer a fundamental depiction description of MDRO. The R (4.1.3, Free Software Foundation) package, specifically the “ggplot2” package, was employed to visualize the temporal and spatial distribution of various multidrug-resistant organisms across 30 provinces in Chine from 2014 to 2020. The Mann–Kendall Trend Test was employed to examine the temporal changes in the proportion of isolation trends for 13 types of MDROs, in order to determine if there was a consistent increase or decrease over time. We employed Stata/SE 15.0(15.0, Stata Corp.) to develop a panel data quantile regression model. The response variables were defined as the isolation proportions of various MDROs. The inclusion of the number of beds in medical and health institutions per 1,000 people, the hospital bed utilization rate, and the average length of stay as explanatory variables, along with the rate of antimicrobial drug use as covariates, is justified due to widely accepted understanding that the rate of antimicrobial drug use has an impact on the occurrence of MDROs. In order to assess sensitivity analysis by incorporating two additional explanatory variables: the average daily burden of bed days and the medical practitioners per 1,000 people.

### Model construction

Panel data refers to a type of two-dimensional data that encompasses both time and individual observations. The panel data model effectively mitigates design and estimation biases by integrating the advantages of cross-sectional and time-series data. The general form of it is as follows.

1 $${y}_{it}={x}_{it}\beta +{z}_{i}\alpha +{\varepsilon }_{it}$$

where ${y}_{it}$ is the value of the explained variable at cross-section $i$ and time $t$; ${x}_{it}$ is the value of the explanatory variable at cross-section $i$ and time $t$; ${\varepsilon }_{it}$ is a random error term, ${z}_{i}\alpha$ is heterogeneity or individual effect, where ${z}_{i}$ contains a constant observable term and a series of group variables that may not change over time. Quantile regression of panel data was employed to estimate the parameters of the panel data model using the quantile regression method. This approach effectively measures the impact of extreme values. The analysis of the impact of explanatory variables on the conditional distribution of explained variables can be enhanced by employing this method at various sub-points. Simultaneously, we implemented measures to control for individual heterogeneity, particularly in order to capture the unique characteristics found in the extreme end of the distribution. In this study, a fixed-effect panel quantile regression model was employed to control for the potential confounding effects of different years on the association between multidrug-resistant organisms.

2 $${Q}_{{y}_{it}}\left(\tau \left|{x}_{it}\right.\right)={\alpha }_{i}+{X}_{it}^{T}\beta \left(\tau \right)+{\mu }_{it},i=1, 2, 3, \dots , N, t=1, 2, 3, \dots , T$$

where the subscript $i$ represents the province (city, autonomous region), $t$ represents the year, $\tau$(0 < $\tau$< 1) indicates the selected decimal point, ${y}_{it}$ is the explained variable in $i$th provinces (cities, autonomous regions) in year $t$, ${Q}_{yit}\left(\tau \left|{x}_{it}\right.\right)$ denotes the τ conditional quantile of ${y}_{it}$ under a given clause ${x}_{it}$; ${\alpha }_{i}$ represents the fixed effect of the $i$th province (city, autonomous region), which does not change with time; ${X}_{it}^{T}$ represents the explanatory variable vector of the $i$th province (city, autonomous region) in year $t$; $\beta$ represents the parameter vector to be estimated, which is assumed not to change with time. ${\mu }_{it}$ indicates the random error term. In this study, five quantile points of 0.1, 0.3, 0.5, 0.7, and 0.9 were selected to construct the regression model.

### Statement

No human participants were involved during study.

### Supplementary Information

Supplementary Information.

## Data Availability

The datasets generated during and/or analyzed during the current study are available from the corresponding author upon request.

## References

[1] Magiorakos AP (2012). Multidrug-resistant, extensively drug-resistant and pandrug-resistant bacteria: An international expert proposal for interim standard definitions for acquired resistance. Clin. Microbiol. Infect..

[2] Arias CA, Murray BE (2009). Antibiotic-resistant bugs in the 21st century–a clinical super-challenge. N. Engl. J. Med..

[3] Price L (2018). Effectiveness of national and subnational infection prevention and control interventions in high-income and upper-middle-income countries: A systematic review. Lancet Infect. Dis.

[4] Jernigan JA (2020). Multidrug-resistant bacterial infections in US hospitalized patients, 2012–2017. N. Engl. J. Med..

[5] Lee YJ (2015). Impact of active screening for methicillin-resistant Staphylococcus aureus (MRSA) and decolonization on MRSA infections, mortality and medical cost: a quasi-experimental study in surgical intensive care unit. Crit. Care (London, England).

[6] Johnson PD (2005). Efficacy of an alcohol/chlorhexidine hand hygiene program in a hospital with high rates of nosocomial methicillin-resistant Staphylococcus aureus (MRSA) infection. Med. J. Aust..

[7] Pittet D (2000). Effectiveness of a hospital-wide programme to improve compliance with hand hygiene. Infection Control Programme. Lancet (London, England).

[8] Lee YJ (2015). Impact of active screening for methicillin-resistant Staphylococcus aureus (MRSA) and decolonization on MRSA infections, mortality and medical cost: A quasi-experimental study in surgical intensive care unit. Crit. Care (Lond., Engl.).

[9] Robotham JV (2011). Screening, isolation, and decolonisation strategies in the control of meticillin resistant Staphylococcus aureus in intensive care units: cost effectiveness evaluation. BMJ.

[10] Balkhy HH, Perl TM, Arabi YM (2016). Preventing healthcare-associated transmission of the Middle East Respiratory Syndrome (MERS): Our Achilles heel. J. Infect. Public Health.

[11] Cowling BJ (2015). Preliminary epidemiological assessment of MERS-CoV outbreak in South Korea, May to June 2015. Euro Surveill..

[12] Park HY (2015). Epidemiological investigation of MERS-CoV spread in a single hospital in South Korea, May to June 2015. Euro Surveill..

[13] Hui DS (2013). Severe acute respiratory syndrome (SARS): Lessons learnt in Hong Kong. J. Thorac. Dis..

[14] Andersen BM (2002). Spread of methicillin-resistant *Staphylococcus*
*aureus* in a neonatal intensive unit associated with understaffing, overcrowding and mixing of patients. J. Hosp. Infect..

[15] Leistner R (2013). The impact of staffing on central venous catheter-associated bloodstream infections in preterm neonates—Results of nation-wide cohort study in Germany. Antimicrob. Resist. Infect. Control.

[16] Clements A (2008). Overcrowding and understaffing in modern health-care systems: Key determinants in meticillin-resistant *Staphylococcus*
*aureus* transmission. Lancet. Infect. Dis..

[17] Haenen A (2022). Hand hygiene compliance and its drivers in long-term care facilities; observations and a survey. Antimicrob Resist Infect Control.

[18] Weissman JS (2007). Hospital workload and adverse events. Med Care.

[19] Cunningham JB, Kernohan WG, Sowney R (2005). Bed occupancy and turnover interval as determinant factors in MRSA infections in acute settings in Northern Ireland: 1 April 2001 to 31 March 2003. J. Hosp. Infect..

[20] Zhang M, Ge J, Lin Z (2020). The impact of the number of hospital beds and spatial heterogeneity on an SIS epidemic model. Acta Appl. Math..

[21] Kaier K (2012). Economic implications of the dynamic relationship between antibiotic use and hospital-acquired infections. Value Health.

[22] Orendi J (2008). Health-care organisation, hospital-bed occupancy, and MRSA. Lancet (London, England).

[23] Jones DR (2011). Hospital bed occupancy demystified and why hospitals of different size and complexity must run at different average occupancy. Br. J. Healthc. Manag..

[24] Kaier K, Mutters NT, Frank U (2012). Bed occupancy rates and hospital-acquired infections—should beds be kept empty?. Clin. Microbiol. Infect..

[25] Fischer D (2019). Overcrowding in a neonatal intermediate care unit: Impact on the incidence of multidrug-resistant gram-negative organisms. BMC Infect. Dis..

[26] Borg MA (2003). Bed occupancy and overcrowding as determinant factors in the incidence of MRSA infections within general ward settings. J. Hosp. Infect..

[27] Hu F, Zhu D, Wang F, Wang M (2018). Current status and trends of antibacterial resistance in China. Clin. Infect. Dis..

[28] Hu FP, Guo Y, Zhu DM, Wang F, Jiang XF, Xu YC, Zhang XJ, Zhang CX, Ji P, Xie Y, Kang M, Wang CQ, Wang AM, Xu YH, Shen JL, Sun ZY, Chen ZJ, Ni YX, Sun JY, Chu YZ, Tian SF, Hu ZD, Li J, Yu YS, Lin J, Shan B, Du Y, Han Y, Guo S, Wei LH, Wu L, Zhang H (2016). Kong Resistance trends among clinical isolates in China reported from CHINET surveillance of bacterial resistance, 2005–2014. Clin. Microbiol. Infect..

[29] Bahadori M (2014). Factors affecting intensive care units nursing workload. Iran. Red Crescent Med. J..

[30] Yoon YK (2016). Current status of personnel and infrastructure resources for infection prevention and control programs in the Republic of Korea: A national survey. Am. J. Infect. Control.

