# Supplementary material for: Multidrug-resistant organisms may be associated with bed allocation and utilization efficiency in healthcare institutions, based on national monitoring data from China (2014–2020)
Source: Sci Rep. 2023 Dec 12;13:22055. doi: 10.1038/s41598-023-49548-6 (PMC10716176; doi:10.1038/s41598-023-49548-6)
Supplement: Supplementary file 1 — Supplementary Information. [file 41598_2023_49548_MOESM1_ESM.pdf]

Multidrug-resistant bacteria may be associated with bed allocation and utilization efficiency in healthcare institutions, based on national monitoring data from China (2014–2020)

Xing-Tian Wang[1,6], Hua Meng[2,6], Dong-Feng Pan[3], Xiao-Yu Zheng[1], Wen-Wen Lu[2], Chen Chen[4], Ming Su[5], Hua Meng[2], Xin-Ya Su[2], Zhuo Liu[2], Xiao-Juan Ma[2], Pei-Feng Liang[4*].

[1]Ningxia Chinese medicine Research Center, Yinchuan, Ningxia Hui Autonomous Region, 750021, China.
[2]School of public health and management, Ningxia Medical University, Yinchuan, Ningxia Hui Autonomous Region, 750004, China.
[3]Department of Emergency Medicine, People's hospital of Ningxia Hui Autonomous region, Yinchuan, Ningxia Hui Autonomous Region, 750002, China.
[4]Department of Medicine statistics, People's Hospital of Ningxia Hui Autonomous Region, Yinchuan, Ningxia Hui Autonomous Region, 750002, China.
[5]Yinchuan Stomatology Hospital, Yinchuan, Ningxia Hui Autonomous Region, 750002, China.
[6]These authors contributed equally: Xing-Tian Wang and Hua Meng.
* E-mail: doctor_pf@126.com.

Supplementary Table1 Statistical description of multidrug-resistant organisms

	Observations	Mean (%)	Std. deviation (%)	Median (%)	lower quartile (%)	upper quartile (%)	Minimum value (%)	Maximum value (%)
MRSA	210	31.64	8.42	32.35	25.53	36.60	15.20	49.00
MRCNS	210	75.91	5.11	76.90	73.38	79.63	56.90	84.30
VREA	210	0.52	0.45	0.40	0.20	0.70	0.00	2.40
VREM	210	1.50	1.73	1.10	0.40	1.83	0.00	11.20
PRSP	210	2.94	2.80	1.90	1.00	3.90	0.00	19.00
ERSP	210	93.65	4.14	95.05	92.08	96.43	72.80	98.80
CTX/CRO-R ECO	210	55.05	5.43	54.70	50.98	58.50	44.90	74.40
CR-ECO	210	1.59	0.83	1.40	1.00	2.10	0.20	5.70
QNR-ECO	210	53.10	6.20	52.10	48.38	57.50	42.10	67.60
CTX/CRO-R KPN	210	32.82	9.35	32.75	26.40	39.10	10.20	58.10
CR-KPN	210	8.29	6.81	5.95	3.10	11.83	0.30	32.80
CR-PAE	210	19.93	6.43	19.15	14.93	25.43	5.70	36.40
CR-ABA	210	54.89	11.66	55.55	48.95	61.25	18.20	82.10

MRSA: methicillin-resistant *Staphylococcus aureus*, MRCNS: Methicillin-resistant coagulase-negative *Staphylococcus*, VREA: vancomycin-resistant *Enterococcus faecalis*, VREM: vancomycin-resistant *Enterococcus faecium*, PRSP: penicillin-resistant *Streptococcus pneumoniae*, ERSP: erythromycin-resistant *Streptococcus pneumoniae*, CTX/CRO-R ECO: cefotaxime or ceftriaxone resistant *Escherichia coli*, CR-ECO: carbapenem-resistant *E. coli*, QNR-ECO: quinolone-resistant *Escherichia coli*, CTX/CRO-R KPN: cefotaxime or ceftriaxone resistant *Klebsiella pneumoniae* , CR-KPN: carbapenem-resistant *Klebsiella pneumoniae*, CR-PAE: carbapenem-resistant *Pseudomonas aeruginosa*, CR-ABA: carbapenem-resistant *Acinetobacter baumannii*.

Supplementary Table 2 Statistical description of health resource indicators

Variables	Observations	Mean	Std. deviation	Median	Lower quartile	Upper quartile	Minimum value	Maximum value
Number of beds in medical and health institutions per 1,000 population	210	5.71	0.92	5.69	5.08	6.37	3.78	7.95
Hospital bed utilization rate (%)	210	87.98	7.80	89.80	83.90	92.70	47.70	100.80
Average length of stay (days)	210	9.52	0.72	9.40	8.98	10.00	8.30	11.40
Utilization rate of antibiotics (%)	210	39.78	7.11	40.40	37.28	43.45	0.00	55.50

Supplementary Table 3 Association between the isolation rate of multidrug-resistant organisms and the health-resource allocation with the panel quantile regression model

MRSA

Variable	Quantile 0.1	Quantile 0.3	Quantile 0.5	Quantile 0.7	Quantile 0.9
Constant	-1.985 (-0.13)	5.334 (-0.40)	9.368 (-0.60)	-13.69 (-0.78)	-8.682 (-0.57)
Number of beds in medical and health	-1.721 (-1.30)	-2.116* (-1.90)	-2.885** (-2.21)	-1.724 (-1.17)	-1.361 (-1.07)

institutions per 1,000 population					
Average daily burden of bed days	-0.393 (-0.09)	2.023 (-0.56)	-0.183 (-0.04)	-5.712 (-1.18)	-1.818 (-0.44)
Hospital bed utilization rate	0.243 (-1.51)	0.331** (-2.43)	0.326** (-2.05)	0.528*** (-2.93)	0.468*** (-3.01)
Medical practitioners per 1,000 population	0.190 (-0.06)	3.100 (-1.11)	1.425 (-0.44)	-2.277 (-0.62)	0.804 (-0.25)
Average hospital stay	-1.051 (-0.98)	-2.031** (-2.23)	-0.227 (-0.21)	2.361* (-1.96)	1.382 (-1.33)
Utilization rate of antibiotics	0.579*** (-5.10)	0.306*** (-3.19)	0.229** (-2.04)	0.242* (-1.90)	0.171 (-1.56)
pseudo R^2	0.1681	0.1627	0.1317	0.1127	0.1932

MRCNS

Variable	Quantile 0.1	Quantile 0.3	Quantile 0.5	Quantile 0.7	Quantile 0.9
Constant	27.410 (-1.27)	30.570*** (-2.89)	45.470*** (-5.95)	60.570*** (-8.26)	66.95*** (-10.65)
Number of beds in medical and health institutions per 1,000 population	0.804 (-0.44)	-0.486 (-0.55)	-1.134*(-1.77)	-1.129*(-1.84)	-1.556*** (-2.95)
Average daily burden of bed days	-3.361 (-0.57)	0.932 (-0.32)	1.455 (-0.70)	0.65 (-0.32)	3.175* (-1.84)
Hospital bed utilization rate	0.597*** (-2.7)	0.397*** (-3.67)	0.215*** (-2.75)	0.129* (-1.73)	0.070 (-1.08)
Medical practitioners per 1,000 population	-1.423 (-0.31)	1.21 (-0.55)	1.963 (-1.23)	1.065 (-0.69)	2.111 (-1.60)
Average hospital stay	0.409 (-0.28)	0.927 (-1.28)	1.151** (-2.20)	0.442 (-0.88)	0.596 (-1.39)
Utilization rate of antibiotics	-0.158 (-1.02)	-0.055 (-0.72)	-0.004 (-0.07)	0.124** (-2.35)	-0.035 (-0.78)
pseudo R^2	0.1584	0.1538	0.1368	0.1340	0.1545

VREA

Variable	Quantile 0.1	Quantile 0.3	Quantile 0.5	Quantile 0.7	Quantile 0.9
Constant	-0.737** (-2.07)	-1.187***(-3.04)	-1.420** (-2.20)	-0.308 (-0.31)	0.621 (-0.38)
Number of beds in medical and health institutions per 1,000 population	-0.186*** (-6.24)	-0.223*** (-6.81)	-0.255*** (-4.70)	-0.394*** (-4.69)	-0.523*** (-3.77)
Average daily burden of bed days	0.361***(-3.71)	0.588***(-5.50)	0.751***(-4.24)	1.023***(-3.73)	1.394***(-3.08)
Hospital bed utilization rate	-0.007* (-1.92)	-0.013*** (-3.30)	-0.019*** (-2.81)	-0.030*** (-2.91)	-0.042** (-2.47)
Medical practitioners per 1,000 population	0.244*** (-3.27)	0.445*** (-5.43)	0.511*** (-3.77)	0.716*** (-3.41)	0.911*** (-2.63)
Average hospital stay	0.085*** (-3.48)	0.107*** (-4.00)	0.168*** (-3.81)	0.168** (-2.45)	0.183 (-1.62)
Utilization rate of antibiotics	0.009*** (-3.42)	0.011*** (-3.99)	0.009** (-1.97)	0.003(-0.40)	-0.001 (-0.12)
pseudo R^2	0.1509	0.1750	0.1618	0.1688	0.2407

VREM

Variable	Quantile 0.1	Quantile 0.3	Quantile 0.5	Quantile 0.7	Quantile 0.9
Constant	-1.518 (-1.47)	-2.513* (-1.94)	-4.525** (-2.29)	-8.029** (-2.35)	-16.310*** (-3.94)
Number of beds in medical and health institutions per 1,000 population	-0.312*** (-3.61)	-0.384*** (-3.53)	-0.550*** (-3.32)	-1.222*** (-4.27)	-0.841** (-2.43)
Average daily burden of bed days	0.458 (-1.62)	0.350 (-0.99)	1.056* (-1.95)	2.612*** (-2.79)	1.567 (-1.38)
Hospital bed utilization rate	-0.001 (-0.14)	0.016(-1.19)	0.006 (-0.28)	-0.017 (-0.48)	0.025 (-0.58)
Medical practitioners per 1,000 population	0.374* (-1.73)	0.215 (-0.79)	1.079*** (-2.6)	3.474*** (-4.85)	3.213*** (-3.70)
Average hospital stay	0.216*** (-3.07)	0.305*** (-3.44)	0.335** (-2.48)	0.403* (-1.73)	1.190*** (-4.21)
Utilization rate of antibiotics	-0.006 (-0.79)	-0.003 (-0.37)	0.004 (-0.31)	0.014(-0.57)	0.003(-0.10)
pseudo R^2	0.0752	0.1303	0.1384	0.1767	0.4270

PRSP

Variable	Quantile 0.1	Quantile 0.3	Quantile 0.5	Quantile 0.7	Quantile 0.9

Constant	-0.599 (-0.34)	0.219 (-0.09)	-2.699 (-0.66)	-4.087 (-0.60)	-17.280** (-2.27)
Number of beds in medical and health institutions per 1,000 population	-0.271* (-1.84)	-0.483** (-2.28)	-0.671* (-1.96)	-1.024* (-1.79)	-1.331** (-2.09)
Average daily burden of bed days	0.532 (-1.11)	0.547 (-0.79)	0.918 (-0.82)	-0.728 (-0.39)	-0.582 (-0.28)
Hospital bed utilization rate	0.015(-0.81)	0.007 (-0.27)	0.010 (-0.25)	0.070 (-1.00)	0.083 (-1.07)
Medical practitioners per 1,000 population	-0.027 (-0.07)	0.412 (-0.78)	0.510 (-0.60)	-0.639 (-0.45)	0.240 (-0.15)
Average hospital stay	-0.041 (-0.35)	-0.008 (-0.04)	0.514* (-1.84)	1.099** (-2.35)	2.473*** (-4.75)
Utilization rate of antibiotics	0.016 (-1.23)	0.025 (-1.38)	-0.009 (-0.32)	0.012 (-0.24)	0.039 (-0.70)
pseudo R^2	0.0740	0.0585	0.0635	0.1208	0.2169

ERSP

Variable	Quantile 0.1	Quantile 0.3	Quantile 0.5	Quantile 0.7	Quantile 0.9
Constant	73.470*** (-3.93)	76.170*** (-8.23)	86.000***(13.46)	89.150**(-26.08)	83.970*** (-23.80)
Number of beds in medical and health institutions per 1,000 population	2.803* (-1.79)	1.658** (-2.14)	0.581 (-1.09)	0.713** (-2.49)	0.287 (-0.97)
Average daily burden of bed days	-11.590** (-2.27)	-4.773* (-1.88)	-1.672 (-0.96)	-1.701* (-1.82)	-0.405 (-0.42)
Hospital bed utilization rate	0.306 (-1.60)	0.152 (-1.61)	0.074(-1.13)	0.059* (-1.70)	0.055 (-1.52)
Medical practitioners per 1,000 population	-3.178 (-0.81)	-0.600(-0.31)	0.405 (-0.30)	-0.149 (-0.21)	1.524** (-2.06)
Average hospital stay	0.457 (-0.36)	0.431 (-0.68)	-0.118 (-0.27)	-0.106 (-0.45)	0.019(-0.08)
Utilization rate of antibiotics	0.098 (-0.73)	0.066 (-1.00)	0.089* (-1.93)	0.078*** (-3.15)	0.109*** (-4.27)
pseudo R^2	0.1565	0.1105	0.0759	0.0618	0.0459

CTX/CRO-R ECO

Variable	Quantile 0.1	Quantile 0.3	Quantile 0.5	Quantile 0.7	Quantile 0.9
Constant	34.270*** (-4.69)	24.520***(-3.23)	8.406 (-0.88)	13.000 (-1.27)	45.980*** (-4.12)
Number of beds in medical and health institutions per 1,000 population	-2.669*** (-4.36)	-2.376*** (-3.73)	-1.275 (-1.59)	-1.232 (-1.44)	-2.579*** (-2.76)
Average daily burden of bed days	8.225*** (-4.11)	7.591*** (-3.65)	1.494 (-0.57)	2.604 (-0.93)	1.696 (-0.56)
Hospital bed utilization rate	-0.212*** (-2.83)	-0.079 (-1.02)	0.212** (-2.18)	0.241** (-2.30)	-0.019 (-0.16)
Medical practitioners per 1,000 population	1.858 (-1.21)	3.443** (-2.16)	0.317 (-0.16)	-0.411 (-0.19)	-2.568 (-1.10)
Average hospital stay	1.721*** (-3.44)	1.479*** (-2.85)	2.152*** (-3.30)	2.159*** (-3.09)	3.155*** (-4.14)
Utilization rate of antibiotics	0.203*** (-3.84)	0.194*** (-3.53)	0.253*** (-3.67)	0.102 (-1.39)	0.081 (-1.00)
pseudo R^2	0.2113	0.1759	0.1613	0.1776	0.2545

CR-ECO

Variable	Quantile 0.1	Quantile 0.3	Quantile 0.5	Quantile 0.7	Quantile 0.9
Constant	-2.834*** (-4.33)	-3.184*** (-3.16)	-3.281*** (-2.74)	-4.046* (-1.89)	-5.360*** (-2.93)
Number of beds in medical and health institutions per 1,000 population	0.031 (-0.57)	0.112 (-1.33)	0.025 (-0.25)	-0.109 (-0.61)	-0.155 (-1.01)
Average daily burden of bed days	-0.240 (-1.34)	-0.763*** (-2.77)	-0.713** (-2.18)	-0.326 (-0.56)	0.683 (-1.36)
Hospital bed utilization rate	0.036*** (-5.43)	0.056*** (-5.44)	0.053*** (-4.36)	0.040* (-1.83)	0.003 (-0.18)
Medical practitioners per 1,000 population	0.215 (-1.57)	0.112 (-0.53)	0.201 (-0.80)	0.219 (-0.49)	0.684* (-1.78)
Average hospital stay	0.042 (-0.94)	0.044 (-0.64)	0.118 (-1.44)	0.296** (-2.02)	0.436*** (-3.48)
Utilization rate of antibiotics	0.001(-0.12)	0.000 (-0.04)	0.002 (-0.18)	0.015 (-0.98)	0.032** (-2.41)
pseudo R^2	0.1016	0.1199	0.1503	0.1146	0.1435

QNR-ECO

Variable	Quantile 0.1	Quantile 0.3	Quantile 0.5	Quantile 0.7	Quantile 0.9
Constant	43.350*** (-5.20)	43.98*** (-4.96)	32.550** (-2.48)	22.770* (-1.81)	27.890** (-2.50)
Number of beds in medical and health institutions per 1,000 population	-3.819*** (-5.46)	-3.051*** (-4.11)	-1.511 (-1.37)	-0.243 (-0.23)	0.426 (-0.46)
Average daily burden of bed days	8.452*** (-3.70)	8.047*** (-3.31)	5.341 (-1.49)	0.693 (-0.20)	0.980 (-0.32)
Hospital bed utilization rate	-0.256*** (-3.00)	-0.408*** (-4.50)	-0.334** (-2.49)	-0.086 (-0.67)	-0.190* (-1.67)
Medical practitioners per 1,000 population	6.290*** (-3.60)	6.477*** (-3.48)	4.113 (-1.49)	0.734 (-0.28)	-0.469 (-0.20)
Average hospital stay	1.643*** (-2.88)	2.658*** (-4.39)	4.076*** (-4.54)	4.889*** (-5.69)	4.994*** (-6.56)
Utilization rate of antibiotics	-0.067 (-1.11)	-0.008 (-0.13)	-0.071 (-0.74)	-0.182** (-2.01)	-0.061(-0.75)
pseudo R^2	0.1472	0.1408	0.1544	0.1894	0.2449

CTX/CRO-R KPN

Variable	Quantile 0.1	Quantile 0.3	Quantile 0.5	Quantile 0.7	Quantile 0.9
Constant	-5.500 (-0.25)	13.930 (-1.19)	-9.932 (-0.61)	-26.060* (-1.75)	-29.120 (-0.99)
Number of beds in medical and health institutions per 1,000 population	-0.199 (-0.11)	-2.337** (-2.38)	-2.932** (-2.15)	-0.663 (-0.53)	-1.061 (-0.43)
Average daily burden of bed days	-5.965 (-1.00)	0.186 (-0.06)	1.259 (-0.28)	-6.837* (-1.68)	-7.564 (-0.94)
Hospital bed utilization rate	0.771*** (-3.46)	0.330*** (-2.76)	0.358** (-2.15)	0.601*** (-3.96)	0.597** (-1.98)
Medical practitioners per 1,000 population	-6.736 (-1.47)	0.557 (-0.23)	3.231 (-0.95)	-4.039 (-1.30)	-3.219 (-0.52)
Average hospital stay	-2.092 (-1.40)	-1.342* (-1.68)	0.313 (-0.28)	3.168*** (-3.12)	5.085** (-2.52)
Utilization rate of antibiotics	0.265* (-1.69)	0.248*** (-2.94)	0.370*** (-3.16)	0.234** (-2.19)	0.055 (-0.26)
pseudo R^2	0.1689	0.1618	0.1408	0.1608	0.1623

CR-KPN

Variable	Quantile 0.1	Quantile 0.3	Quantile 0.5	Quantile 0.7	Quantile 0.9
Constant	-18.830***(-2.90)	-31.640***(-4.88)	-31.020***(-3.18)	-28.940** (-2.27)	3.458 (-0.10)
Number of beds in medical and health institutions per 1,000 population	0.242 (-0.44)	-0.115 (-0.21)	-1.034 (-1.26)	0.289 (-0.27)	2.322 (-0.78)
Average daily burden of bed days	-0.609 (-0.34)	0.660 (-0.37)	4.420* (-1.65)	-2.917 (-0.84)	-13.720 (-1.40)
Hospital bed utilization rate	0.227*** (-3.41)	0.282*** (-4.25)	0.179* (-1.79)	0.437*** (-3.36)	0.672* (-1.84)
Medical practitioners per 1,000 population	1.875 (-1.38)	6.627*** (-4.87)	8.356*** (-4.08)	3.440 (-1.29)	-4.086 (-0.55)
Average hospital stay	-0.431 (-0.97)	-0.771* (-1.74)	-0.596 (-0.89)	-0.439 (-0.50)	-2.321 (-0.95)
Utilization rate of antibiotics	0.0438 (-0.93)	0.0945** (-2.02)	0.123* (-1.74)	0.077 (-0.84)	0.106 (-0.41)
pseudo R^2	0.0805	0.1404	0.1681	0.1790	0.1823

CR-PAE

Variable	Quantile 0.1	Quantile 0.3	Quantile 0.5	Quantile 0.7	Quantile 0.9
Constant	-8.204 (-0.59)	-2.224 (-0.23)	-21.840* (-1.93)	-26.830* (-1.90)	-39.040*** (-4.54)
Number of beds in medical and health institutions per 1,000 population	-2.521** (-2.15)	-3.392*** (-4.27)	-3.443*** (-3.63)	-2.828** (-2.39)	-2.043*** (-2.84)
Average daily burden of bed days	2.591 (-0.68)	4.486* (-1.73)	5.586* (-1.80)	4.296 (-1.11)	4.906** (-2.08)
Hospital bed utilization rate	0.138 (-0.97)	0.089 (-0.92)	0.207* (-1.79)	0.252* (-1.74)	0.203** (-2.30)
Medical practitioners per 1,000 population	4.525 (-1.54)	6.597*** (-3.32)	8.849*** (-3.73)	7.058** (-2.38)	5.895*** (-3.27)
Average hospital stay	0.861 (-0.90)	0.182 (-0.28)	0.522 (-0.67)	1.532 (-1.59)	2.885*** (-4.91)
Utilization rate of antibiotics	-0.012(-0.12)	0.082 (-1.21)	0.127 (-1.56)	0.076 (-0.75)	0.197*** (-3.18)
pseudo R^2	0.0949	0.1643	0.1944	0.2206	0.2501

CR-ABA					
**Variable**	**Quantile 0.1**	**Quantile 0.3**	**Quantile 0.5**	**Quantile 0.7**	**Quantile 0.9**
Constant	-71.580** (-1.98)	-47.330** (-2.18)	-22.410* (-1.66)	-24.520 (-1.28)	-13.180 (-0.53)
Number of beds in medical and health institutions per 1,000 population	1.035（-0.34)	1.682 (-0.92)	-0.0461 (-0.04)	-0.711 (-0.44)	-1.376 (-0.66)
Average daily burden of bed days	5.853 (-0.59)	-0.040 (-0.01)	2.311 (-0.63)	5.586 (-1.06)	12.270* (-1.79)
Hospital bed utilization rate	0.875** (-2.37)	0.624*** (-2.81)	0.465*** (-3.38)	0.210 (-1.07)	0.033 (-0.13)
Medical practitioners per 1,000 population	0.361 (-0.05)	1.382 (-0.3)	2.373 (-0.84)	3.630 (-0.90)	4.324 (-0.82)
Average hospital stay	-0.003 (-0.00)	2.613* (-1.76)	2.325** (-2.53)	4.681*** (-3.57)	4.594*** (-2.68)
Utilization rate of antibiotics	0.328 (-1.26)	0.146 (-0.93)	0.104 (-1.07)	0.105 (-0.76)	0.021 (-0.12)
pseudo R^2	0.2085	0.1200	0.1166	0.1109	0.1914

* p<0.05, ** p<0.01, *** p<0.001, The value is "Regression coefficients" in table and "t" in brackets. MRSA: methicillin-resistant *Staphylococcus aureus*, MRCNS: Methicillin-resistant coagulase-negative *Staphylococcus*, VREA: vancomycin-resistant *Enterococcus faecalis*, VREM: vancomycin-resistant *Enterococcus faecium*, PRSP: penicillin-resistant *Streptococcus pneumoniae*, ERSP: erythromycin-resistant *Streptococcus pneumoniae*, CTX/CRO-R ECO: cefotaxime or ceftriaxone resistant *Escherichia coli*, CR-ECO: carbapenem-resistant *E. coli*, QNR-ECO: quinolone-resistant *Escherichia coli*, CTX/CRO-R KPN: cefotaxime or ceftriaxone resistant *Klebsiella pneumoniae* , CR-KPN: carbapenem-resistant *Klebsiella pneumoniae*, CR-PAE: carbapenem-resistant *Pseudomonas aeruginosa*, CR-ABA: carbapenem-resistant *Acinetobacter baumannii*.
